# Properties of the vastus lateralis muscle in relation to age and physiological function in master cyclists aged 55–79 years

**DOI:** 10.1111/acel.12735

**Published:** 2018-03-08

**Authors:** Ross D. Pollock, Katie A. O'Brien, Lorna J. Daniels, Kathrine B. Nielsen, Anthea Rowlerson, Niharika A. Duggal, Norman R. Lazarus, Janet M. Lord, Andrew Philp, Stephen D. R. Harridge

**Affiliations:** ^1^ Centre of Human and Aerospace Physiological Sciences King's College London London UK; ^2^ MRC‐Arthritis Research UK Centre for Musculoskeletal Ageing Research Institute of Inflammation and Ageing University of Birmingham Birmingham UK; ^3^ School of Sport, Exercise and Rehabilitation Sciences University of Birmingham Birmingham UK; ^4^ Diabetes and Metabolism Division Garvan Institute of Medical Research Darlinghurst Australia

**Keywords:** aging, ATPase histochemistry, immunohistochemistry, master athletes, mitochondria, muscle

## Abstract

In this study, results are reported from the analyses of *vastus lateralis* muscle biopsy samples obtained from a subset (*n* = 90) of 125 previously phenotyped, highly active male and female cyclists aged 55–79 years in regard to age. We then subsequently attempted to uncover associations between the findings in muscle and in vivo physiological functions. Muscle fibre type and composition (ATPase histochemistry), size (morphometry), capillary density (immunohistochemistry) and mitochondrial protein content (Western blot) in relation to age were determined in the biopsy specimens. Aside from an age‐related change in capillary density in males (*r* = −.299; *p* = .02), no other parameter measured in the muscle samples showed an association with age. However, in males type I fibres and capillarity (*p* < .05) were significantly associated with training volume, maximal oxygen uptake, oxygen uptake kinetics and ventilatory threshold. In females, the only association observed was between capillarity and training volume (*p* < .05). In males, both type II fibre proportion and area (*p* < .05) were associated with peak power during sprint cycling and with maximal rate of torque development during a maximal voluntary isometric contraction. Mitochondrial protein content was not associated with any cardiorespiratory parameter in either males or females (*p* > .05). We conclude in this highly active cohort, selected to mitigate most of the effects of inactivity, that there is little evidence of age‐related changes in the properties of VL muscle across the age range studied. By contrast, some of these muscle characteristics were correlated with in vivo physiological indices.

## 1 INTRODUCTION

Aging is associated with declines in multiple physiological systems (Ferrari, Radaelli & Centola, 2003; Power, Dalton & Rice, 2013; Sharma & Goodwin, 2006). Skeletal muscle in later life is typically characterized by a loss of tissue mass (sarcopenia) and a decline in contractile function (dynapenia) (Power et al., 2013). Anatomically this has been attributed to a selective atrophy of fast‐contracting type II muscle fibres (Andersen, 2003; Narici & Maffulli, 2010; Nilwik et al., 2013) and a reduction in motor unit and muscle fibre number (Hepple & Rice, 2016; Lexell, Henrikssonlarsen, Winblad & Sjostrom, 1983; Piasecki, Ireland, Jones & McPhee, 2016). Furthermore, characteristics of the typical older muscle include a decline in muscle quality as evidenced by infiltrations of fat and connective tissue (Agley, Rowlerson, Velloso, Lazarus & Harridge, 2013; Hogrel et al., 2015) and reduced muscle oxidative capacity, through a decline in mitochondrial number and function (Ghosh et al., 2011; Lanza et al., 2008; Rooyackers, Adey, Ades & Nair, 1996; Short et al., 2005) and muscle capillarity (Coggan et al., 1992). However, because of the complex interaction between aging per se and the decline in physical activity with age in humans, the extent to which these changes in muscle are driven by the underlying biological aging process remains far from clear (Harridge & Lazarus, 2017). Indeed, many of the current perceptions of the relationship between age and physiological function, including musculoskeletal function, are based on results from populations whose physical activity status and lifestyle are low and/or are poorly characterized (Booth & Lees, 2006; Hawkins, Wiswell & Marcell, 2003; Lazarus & Harridge, 2010). As our genetic inheritance stems from a period when high levels of physical activity were the likely norm (Booth & Lees, 2006), it has been argued that being physically active is the default position required for maintaining health and physical function throughout the lifespan (Booth & Lees, 2006; Lazarus & Harridge, 2010).

To overcome these concerns, we (Pollock et al., 2015) recently undertook a comprehensive phenotyping of a cohort of 125 male and female cyclists aged 55–79 years, where training volume was maintained with age, to examine the relationship between age and physiological function. While acknowledging that genetic differences between individuals may influence the aging trajectory, the genotype of the cohort, albeit for only two common “performance” genotypes, was similar to the general population and not skewed to an endurance‐based phenotype (Pollock et al., 2015). Here, we report further results from this cohort of highly active individuals where we have analysed muscle biopsy samples (*n* = 90) obtained from the *vastus lateralis* (VL) muscle. Fibre type composition, fibre sizes, capillary density and mitochondrial protein characteristics were examined in relation to age. Our hypothesis was that in these individuals who showed similar high levels of physical activity (Pollock et al., 2015), any changes associated with age could be ascribed to an inherent aging process not confounded by inactivity. Furthermore, these properties of the VL were also examined in relation to a selection of relevant in vivo physiological characteristics of the participants associated with either aerobic (e.g. VO_2max_) or explosive muscle function.

## 2 RESULTS

### 2.1 Relation between age and vastus lateralis characteristics

**Figure 1:**
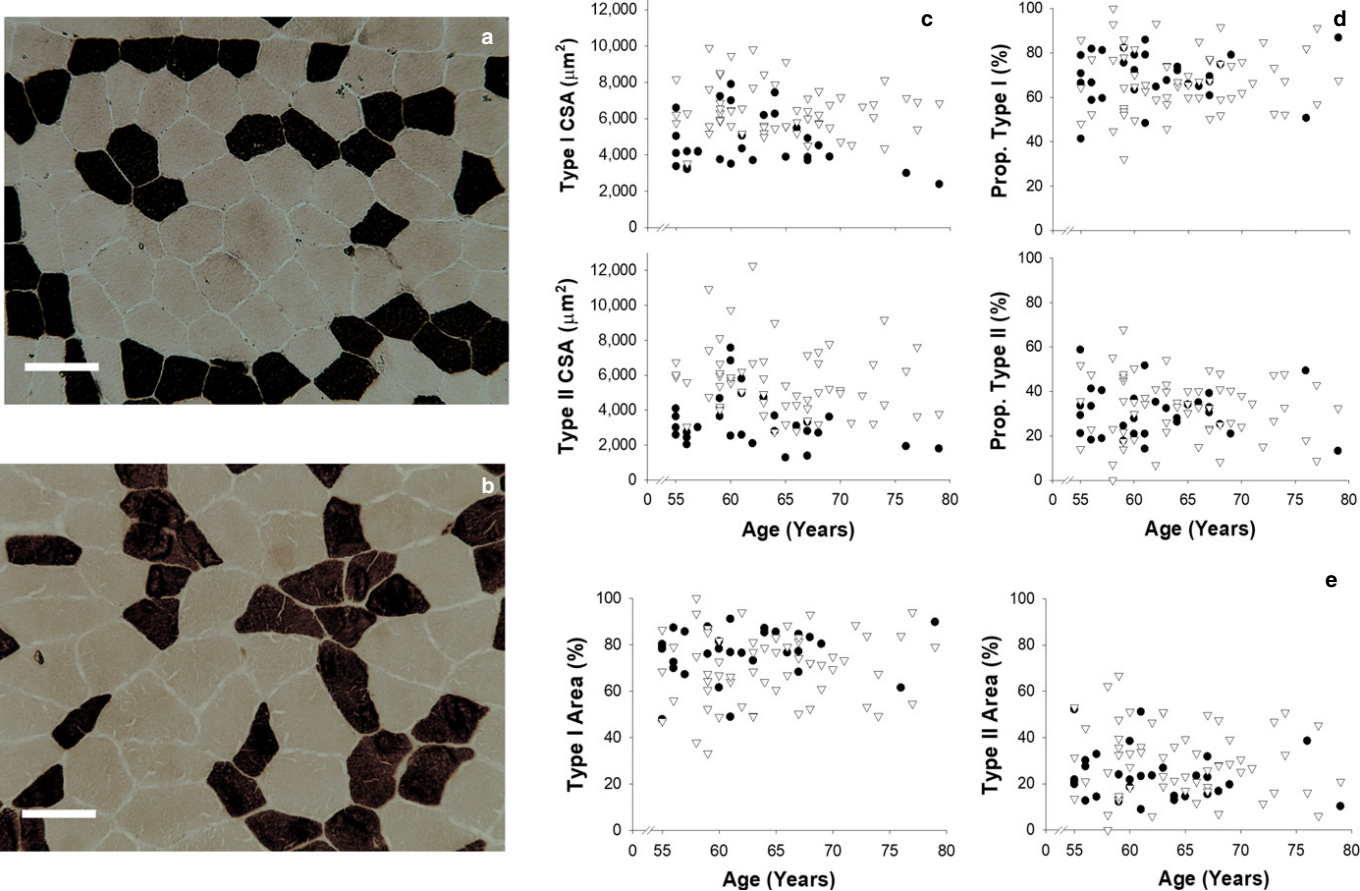
Results from histochemical analysis of sections obtained from muscle biopsies. Panels (a) and (b) display examples of sections obtained from subjects aged 71 (a) and 59 (b) years. A pH of 10.2 was used for section staining. Type I (white) and type II (black) fibres can be seen with the scale bar equating to 50 μm. Panel (c) shows the relationship between age and the cross‐sectional area (CSA) of type I (upper) and type II (lower) fibres. Panel (d) shows the relationship between age and the proportion of type I (upper) and type II (lower) fibres within the VL. Panel (e) shows the relationship between age and the percentage area of the muscle taken up by type I (left) and type II (right) fibres. Data are displayed for males (▽) and females (●) separately

Examples of the ATPase staining for a 71‐ and 59‐year‐old individual are shown in Figure 1a. The examples shown are representative of the entire cohort. In general, the ATPase staining revealed regularly shaped polyhedral cells with a morphology similar to that expected in a healthy young individual. Overall, there were negligible numbers of type IIx and IIc fibres as indicated by an average of 1 fibre identified per subject. For the cohort as a whole, the proportion of fibre types was typical of a slower phenotype, with 68.3 ± 1.2% type I fibres, and the fast fibres being almost exclusively type IIA. For both sexes, there was no age‐related change in the proportion or area of type I or II fibres (Figure 1d–e; *p* > .05 in all cases) and no effect of age on the CSA of either type I or II muscle fibres (Figure 1c; *p* > .05).

**Figure 2:**
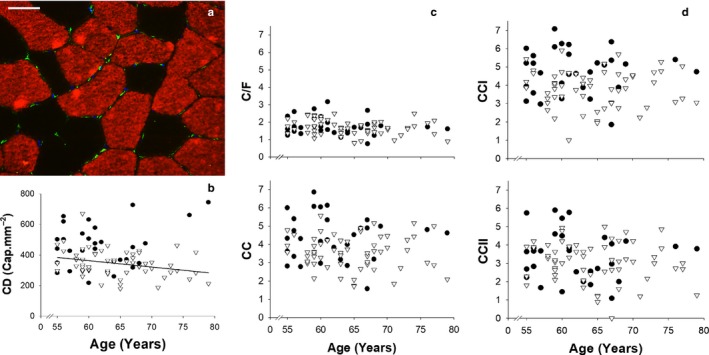
Results obtained from the immunohistochemical staining for capillaries. Panel (a) displays an example stain from a subject aged 60 years. Fibres expressing MHC‐I are red and capillaries are green while the scale bar is equivalent to 50 μm. Panel (b) shows the relationship between capillary density and age. The black line indicates a negative correlation between capillary density and age in males (*y* = −4.18*x* + 614.68; *r* ^2^ = .089). Panel (c) shows the relationship between age and capillary‐to‐fire ratio (C/F; upper) and capillary contacts per fibre (CC; lower). Panel (d) show shows the relationship between age and the average number of capillary contacts per type I (CCI; upper) and II (CCII; lower) fibres. Data are displayed for males (▽) and females (●) separately

With regard to capillarity, there was a linear decline in capillary density (Figure 2b) with age in males (*r* = −.299; *p* = .02), but not in females (*p* > .05). There was no relationship between age and capillary‐to‐fibre ratio, capillary contacts and capillary contacts of type I and II fibres in either gender (Figure 2c–d; *p* > .05 in all cases).

**Figure 3:**
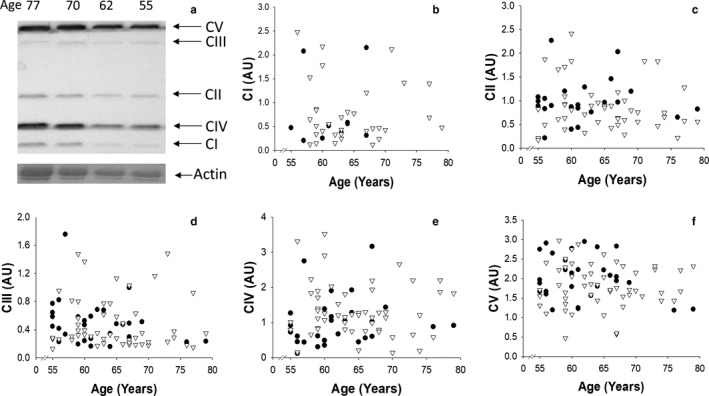
Results obtained from Western blot analysis of mitochondrial protein content from muscle biopsies obtained from the VL. Panel (a) shows an example Western blot with mitochondrial protein regions highlighted, alongside the actin band displayed through a Ponceau S stain. The relationship between total protein content, corrected to actin, of complexes I, II, III, IV and V and age is shown in panels (b–f), respectively. Data are displayed for males (▽) and females (●) separately. MWM = molecular weight marker; CI = mitochondrial complex I; CII = mitochondrial complex II; CIII = mitochondrial complex III; CIV = mitochondrial complex IV; CV = mitochondrial complex V

Western blotting analysis of mitochondrial complex proteins (CI, CII, CIII, CIV and CV) revealed no age‐related effects in either males or females (*p* > .05 in all cases; Figure 3).

### 2.2 Relation between characteristics of the VL and physiological function

Given that little or no association was observed between properties of the biopsy samples and age, the properties of the muscle were evaluated in relation to physiological characteristics of the participants, as previously reported (Pollock et al., 2015), irrespective of age.

A summary of the mean values obtained for each physiological function assessed is shown in Table 1, and the results of the correlation analysis (*r*‐ and *p*‐values) are summarized in Table 2 and Table 3.

**Table 1:** Mean muscle characteristics and physiological parameters for each variable assessed

	Male	Female
Type I CSA (μm^2^)	6451.8 (174.9)	4709.1 (263.4)^a^
Type II CSA (μm^2^)	5624.9 (258.4)	3344.2 (270.3)^a^
Prop. Type I (%)	67.7 (1.2)	69.6 (2.0)
Prop. Type II (%)	32.2 (1.8)	30.4 (2.0)
Type I Area (%)	70.5 (2.0)	76.6 (2.0)
Type II Area (%)	29.5 (2.0)	23.4 (2.0)
CD (Cap/mm)	345.0 (11.1)	458.3 (25.7)^a^
C/F	1.67 (0.06)	1.77 (0.10)
CC	3.60 (0.12)	4.39 (0.22)^a^
CCI	3.81 (0.13)	4.78 (0.22)^a^
CCII	3.06 (0.14)	3.44 (0.24)
CI	0.77 (0.11)	0.75 (0.23)
CII	0.87 (0.08)	1.00 (0.09)
CIII	0.49 (0.05)	0.51 (0.06)
CIV	1.34 (0.10)	1.01 (0.13)
CV	1.81 (0.08)	2.03 (0.10)
Monthly cycling distance (km)	753 (46)	551 (39)^a^
VO_2max_ (ml kg^−1^ min^−1^)	45.7 (0.9)	40.7 (1.4)^a^
VO_2max_ (ml [kg FFM]^−1^ min^−1^)	57.8 (1.0)	56.7 (1.5)
VT (ml [kg FFM]^−1^ min^−1^)	43.8 (0.8)	44.4 (1.0)
τ (s)	24.7 (0.8)	22.9 (1.0)
KE torque (Nm/[kg D FFM])	17.2 (0.5)	17.8 (0.8)
RTD1/6 (% MVC/s)	463 (25)	502 (30)
RTD1/2 (% MVC/s)	599 (36)	646 (38)
RTD2/3 (% MVC/s)	416 (29)	566 (42)^a^
PP (W/[kg LL FFM])	53.5 (0.9)	56.2 (1.1)
Vopt (rpm)	97.8 (1.2)	95.0 (1.5)

Data are mean (SE). CD, capillary density; C/F, capillary‐to‐fibre ratio; CC, capillary contacts per fibre; CCI, capillary contacts per type I fibre; CCII, capillary contacts per type II fibre; CI–V, mitochondrial protein content of complexes I–V; FFM, fat‐free mass; D, dominant leg; LL, lower limb; KE, knee extensor; VT, ventilatory threshold; τ, oxygen uptake kinetics; RTD, rate of torque development; PP, peak power.

a Significantly different from males (*p* < .05).

**Table 2:** Results of the correlation analysis between type I muscle fibres, capillarity, mitochondrial protein content and relevant physiological variables

	Monthly cycling distance	VO_2max_	VT	τ
Males				
Proportion	**.195 (0.031)**	**.331 (0.010)**	**.262 (0.043)**	**−.403 (0.002)**
Relative area	**.212 (0.019)**	**.299 (0.020)**	.218 (0.094)	**−.407 (0.002)**
CD	.160 (0.076)	**.316 (0.027)**	**.348 (0.006)**	**−.355 (0.010)**
C/F	**−**.076 (0.570)	**.294 (0.022)**	**.295 (0.022)**	**−**.205 (0.125)
CC	.037 (0.680)	.189 (0.149)	**.304 (0.018)**	**−.294 (0.025)**
CCI	**−**.002 (0.979)	.124 (0.345)	.239 (0.066)	**−.292 (0.026)**
CCII	**−**.034 (0.709)	.150 (0.252)	.233 (0.073)	**−**.093 (0.487)
CI	**.263 (0.032)**	.144 (0.239)	.222 (0.070)	**−**.158 (0.136)
CII	.199 (0.195)	.209 (0.168)	.158 (0.299)	**−**.035 (0.823)
CIII	.192 (0.182)	.046 (0.746)	**−**.020 (0.891)	**−**.079 (0.590)
CIV	.215 (0.130)	.086 (0.543)	.140 (0.321)	**−**.019 (0.898)
CV	**−**.061 (0.667)	.114 (0.415)	.132 (0.340)	.028 (0.843)
Females				
Proportion	.105 (0.580)	.179 (0.345)	**−**.065 (0.733)	**−**.103 (0.422)
Relative area	.021 (0.872)	**−**.034 (0.789)	**−**.002 (0.986)	**−**.048 (0.708)
CD	**.400 (0.029)**	.061 (0.748)	.152 (0.422)	**−**.218 (0.090)
C/F	**.375 (0.041)**	.192 (0.309)	.209 (0.267)	**−**.226 (0.230)
CC	.251 (0.180)	.154 (0.416)	.162 (0.392)	**−**.145 (0.261)
CCI	.250 (0.183)	.146 (0.441)	.171 (0.368)	**−**.131 (0.309)
CCII	.067 (0.724)	.142 (0.455)	.149 (0.432)	.060 (0.643)
CI	**−**.244 (0.325)	**−**.067 (0.788)	.255 (0.369)	.111 (0.655)
CII	**−**.115 (0.584)	**−**.011 (0.959)	**−**.042 (0.841)	**−**.036 (0.863)
CIII	.083 (0.670)	.258 (0.177)	.196 (0.309)	**−**.022 (0.912)
CIV	**−**.002 (0.992)	**−**.261 (0.172)	**−**.206 (0.283)	**−**.050 (0.796)
CV	**−**.109 (0.572)	.126 (0.516)	.186 (0.333)	.078 (0.688)

The results displayed are the correlation coefficient and associated *p*‐value (*r* (*p*)). Significant correlations are shown in bold.

**Table 3:** Results of the correlation analysis between type II muscle fibre characteristics and relevant physiological indices

	KE torque	RTD1/6	RTD1/2	RTD2/3	PP	Vopt
Males						
Proportion	.120 (0.359)	.092 (0.483)	.126 (0.337)	**.174 (0.049)**	**.271 (0.036)**	**−**.188 (0.150)
Relative area	.005 (0.971)	.046 (0.725)	.119 (0.363)	**.211 (0.017)**	**.278 (0.032)**	**−**.138 (0.294)
Females						
Proportion	.236 (0.209)	**−**.149 (0.431)	**−**.183 (0.332)	**−**.174 (0.358)	.226 (0.230)	**−**.145 (0.445)
Relative area	.062 (0.630)	**−**.122 (0.344)	**−**.117 (0.363)	**−**.103 (0.422)	.113 (0.382)	.23 (0.904)

The results displayed are the correlation coefficient and associated *p*‐value (*r* (*p*)). Significant correlations are shown in bold.

The monthly distance cycled by male subjects was weakly but significantly correlated with type I fibre proportion and area (*r* = .195 and .212, respectively, *p* < .05; Table 2). There was a linear relationship between the monthly distance cycled and capillary‐to‐fibre ratio and capillary density in females, while there was only a tendency towards this with capillary density in males (*r* = .160; *p* = .076). A linear relationship existed between the monthly distance cycled and complex I protein content in males only (*r* = .263, *p* = .032; Table 2).

In regard to cardiorespiratory parameters, VO_2max_ and O_2_ uptake kinetics were found to be positively correlated with type I fibre proportion and area in males (Table 2). Ventilatory threshold also increased linearly with the proportion of type I fibres in males. There were no associations between cardiorespiratory parameters and type I fibre characteristics in females (*p* > .05 in all cases).

VO_2max_ linearly increased with capillary density and capillary‐to‐fibre ratio in males (Table 2), while capillary density, capillary‐to‐fibre ratio and capillary contacts all linearly increased with ventilatory threshold in males (Table 2). O_2_ uptake kinetics were negatively correlated with capillary density, capillary contacts and type II fibre capillary contacts (Table 2) in males, while there was a tendency towards this with capillary density in females (*r* = −.218; *p* = .09). No significant associations between capillarity and cardiorespiratory parameters were found in females (*p* > .05).

Mitochondrial complex protein content was not associated with any cardiorespiratory parameter in either males or females (*p* > .05 in all cases).

With regard to strength/power‐related parameters and type II fibre characteristics (Table 3), PP and RTD2/3 were found to be positively, but weakly (*r* = .174 to .278; *p* < .05), associated with type II fibre proportion and area in males. No other associations between strength/power parameters and type II fibre characteristics were found in males or females (*p* > .05).

## 3 DISCUSSION

Our understanding of the aging process is confounded by many factors, including genetic variation and differences in socio‐economic, healthcare and nutritional status and, crucially, the physical activity status of experimental participants (Lazarus & Harridge, 2010; Metter et al., 1999). In many studies, data are obtained from those in whom physiological function is influenced not only by the aging process, but also by an interaction with negative lifestyle factors, including sedentary behaviour (Booth & Lees, 2006; Hawkins et al., 2003; Lazarus & Harridge, 2010). To remove physical inactivity as a confounding factor, we have recently investigated the physiological characteristics of a group of highly active older individuals (Pollock et al., 2015), with this group of cyclists having activity levels considerably above that likely to be required to offset the negative effects of inactivity (Lazarus & Harridge, 2017). It may be possible that an unknown intrinsic factor facilitates the ability of these amateur cyclists to participate in noncompetitive long‐distance events; however, in the context of experimental procedures at our disposal, we have not been able to uncover such a factor.

In our original investigation where studies were confined to in vivo physiological measurements (Pollock et al., 2015), we found that strong associations between age and physiological function were not observed, in males or females, for the majority of variables, including no association between age and appendicular muscle mass (skeletal muscle mass index, SMI) determined from a dual‐energy X‐ray absorptiometry (DXA) scan. The aims of this study were twofold. The first was to focus our observations on the relationship between age and a number of properties (fibre type distribution and sizes, capillarity and mitochondrial complex protein composition) of skeletal muscle analysed from biopsy samples taken from the VL muscle. Here, the results in both males and females revealed no association between the characteristics of the VL and the age of the donor with the exception of capillary density in males. The second was to correlate the properties of the VL with those in vivo physiological indices which could be expected to be influenced by the known characteristics of the different fibre types. Here, a number of associations were observed in males, but not females.

### 3.1 Relationship between muscle properties and age

#### 3.1.1 Fibre type distribution and size

Histological images of sections of the VL processed using ATPase histochemistry (Figure 1) show regularly shaped polyhedral muscle cells with an overall morphology that is typically associated with healthy young individuals rather than older muscle. The morphology of an older muscle would typically be characterized by having type II fibre atrophy (Snijders, Verdijk & van Loon, 2009) and angular fibres, as well as often showing infiltrations of fat and connective tissue (Agley et al., 2013; Andersen, 2003; Hogrel et al., 2015). In our older cyclists with maintained high levels of endurance exercise, the predominance of type I fibres (67.7% and 69.6% for males and females, respectively) was as expected for this endurance‐trained phenotype. We observed no relation between age and fibre type distribution, while the number of type IIc fibres (i.e. those co‐expressing both slow and fast myosin heavy chain (MHC) isoforms) identified histologically was negligible. A higher number of fibres co‐expressing MHC isoforms has been reported in the muscles of older people (Klitgaard et al., 1990aa). In this regard, it has been suggested that while there is a well‐documented decline in type II muscle fibre size, if there is an age‐related conversion of fibre type, then an atrophy of type I fibres may also occur and may have been underestimated in aging populations (Purves‐Smith, Solbak, Rowan & Hepple, 2012). However, in the present investigation, while the female cyclists had significantly smaller fibres than the males, no correlation with age was observed for either sex or for either type I or type II fibres.

This finding is in contrast to a similar cross‐sectional study on active people, who were sprinters not cyclists, aged 18–84 years (Korhonen et al., 2006), where an age‐related loss of type II fibre size was reported. Previously, Klitgaard et al. (1990bb) had shown a maintenance of type II fibre size in the leg muscles of older strength trained males (aged 69 years) compared to the sedentary, but no maintenance of type II fibres in older endurance runners or swimmers. In another study comparing older people who regularly participate in moderate‐to‐vigorous physical activity three times a week (aged 71 years), a decline in type II fibre size was also observed. The lack of consistency in findings across studies may be explained in part by differences in the age ranges studied, exercise type and age‐related changes to training intensity and volume that may occur between cohorts (Pollock, Foster, Knapp, Rod & Schmidt, 1987; Tanaka & Seals, 2003). In the present study, the cohort of cyclists spanned a relatively narrow age range (55–79 years), but no age‐related reduction in reported training volume was reported.

#### 3.1.2 Capillarity

Endurance exercise has a well‐documented effect of improving both local oxygen delivery, through angiogenesis, and oxygen utilization, through mitochondrial biogenesis (Hawley, 2002; Lundby & Jacobs, 2016). In regard to local O_2_ delivery, there was no difference in the number of capillaries between males and females when expressed as number per fibre, but when expressed per unit area, the females had a higher capillary density. This phenomenon could be explained by the significantly smaller fibres of the female cyclists and gives a potential physiological advantage in terms of a lower distance for O_2_ diffusion into contracting muscle fibres. In the females, we observed no association with any measure of capillarity and age, but we did observe a negative correlation between age and density (capillaries per mm^2^) in the males. As muscle fibre size was unchanged with age and in light of a maintained training volume, the declining capillary density in males could thus be attributed to the aging process and not to an interaction of aging with inactivity. However, the *r* ^2^ value for this correlation was low with only 8.9% of the variance in capillary density explained by age. Furthermore, as no other measures of capillarity were found to be associated with age, the physiological relevance of this weak association remains unclear. In a previous study, older trained males were shown to have a similar capillary density to young trained males (Proctor, Sinning, Walro, Sieck & Lemon, 1995). Furthermore, while we cannot fully explain the discrepancy in the capillary density–age association between males and females, it may be related to the sex‐specific role of androgens in angiogenesis and the declining androgen levels typically observed with age (Sieveking et al., 2010).

#### 3.1.3 Mitochondrial complex protein content

In a study of sedentary people, Short et al. (2005) observed an age‐related decline in mitochondrial protein content and ATP production in a cross‐sectional study across a wide age range of participants (18–89 years). Master athletes, however, are known to have superior mitochondrial enzyme activity and aerobic capacity compared to sedentary individuals of the same age (Coggan et al., 1993; Proctor et al., 1995) and also have significantly greater expression of mitochondrial proteins involved in key metabolic processes in skeletal muscle (Lanza et al., 2008). In the present study, we measured the protein concentrations of mitochondrial complexes I–V, but observed no declines as a result of age, suggesting that with high levels of activity, mitochondrial dysfunction with age may be minimal. A dissociation between mitochondrial protein content and mitochondrial respiratory function has recently been reported (Spendiff et al., 2016); however, we were unable to explore this feature because respiratory measurements were not possible on our frozen biopsy samples. In this regard, a recent study by Distefano et al. (2017) reported no age associations with either mitochondrial respiration or protein content, although they were related to the level of cardiorespiratory fitness irrespective of age, a point we explore further below.

### 3.2 Relation between muscle properties and physiological function

#### 3.2.1 Aerobic capability

While acknowledging that multiple physiological processes are involved in whole‐body exercise, the second aim of the study was to investigate the relationship between the properties of the VL muscle and a number of relevant physiological indices we have previously reported (Pollock et al., 2015). Endurance exercise is closely coupled to aerobic metabolism and thus associated with the generic properties of slow‐oxidative, type I muscle fibres. The predominance of type I fibres in the VL muscle of our male and female cyclists was thus not surprising. In the male cyclists, we observed that the proportion of type I fibres and capillarity were associated with a number of in vivo physiological indices pertaining to aerobic exercise function: VO_2max_, ventilatory threshold and oxygen uptake kinetics. These properties of the VL were also associated with training volume to which mitochondrial complex protein I was also associated. These relationships might simply be explained by the known effects of endurance training commonly observed on skeletal muscle oxidative capacity in young adults (Gjøvaag & Dahl, 2008; Hepple, Mackinnon, Goodman, Thomas & Plyley, 1997; Holloszy & Coyle, 1984) and the role that these have in influencing endurance cycling performance and aerobic power (Bassett & Howley, 2000). The findings relating to oxygen uptake kinetics are in general agreement with previous research indicating that the association between oxygen uptake kinetics and type I fibres is in part due to a greater capillarity of type I fibres which reduces the variability in blood flow to, and the transit time through, the working muscle at the onset of exercise (Piiper, 1990; Pringle et al., 2003). Despite the strongest association of the entire study being between oxygen uptake kinetics and type I fibre area, this only explained 17% of the variance in oxygen uptake kinetics, further highlighting the complexity of comparing in vivo physiological function to single in vitro measurements.

In contrast to capillarity, there was no association between mitochondrial protein complex content and in vivo physiological function, although, as discussed above, an association between mitochondrial respiratory capacity and in vivo cardiorespiratory fitness has previously been shown (Distefano et al., 2017). Interestingly, when studying isolated muscle groups, O_2_ delivery to the working muscle has been identified as a main factor limiting VO_2max_ in endurance‐trained individuals (Richardson et al., 1999) in contrast to mitochondrial function in sedentary individuals (Haseler, Lin & Richardson, 2004). This possible difference in delivery and utilization between athletes and the sedentary, coupled with a dissociation between mitochondrial protein content and mitochondrial respiratory function (Spendiff et al., 2016), may contribute to a lack of association between mitochondrial complex protein content and markers of aerobic function in the present study.

In contrast to males, there was no association between any characteristic of the VL and the physiological indices measured in the female cyclists, although a positive association was observed between training volume and capillary density and capillary‐to‐fibre ratio. The differences in male and females with regard to these associations are difficult to explain with certainty, but will be partly influenced by the higher *r* ^2^ required to achieve statistical significance in the lower number of females (*n* = 30) studied compared to males (*n* = 60).

#### 3.2.2 In vivo muscle function

In contrast to endurance exercise, explosive physical activities require the rapid generation of force and high power outputs. These are properties of muscle that are associated with the faster molecular motor of type IIa and IIx MHC isoform‐containing fibres (Bottinelli, Canepari, Pellegrino & Reggiani, 1996; Harridge et al., 1996; Pearson, Cobbold, Orrell & Harridge, 2006) and highlight the additional problem for power generation for many older people caused by a selective atrophy of fast‐contracting type II fibres. In this regard, in the male cyclists both type II fibre proportion and area were associated with peak power output during sprint cycling (normalized to lower limb muscle mass) and with the rate of torque development during a maximum voluntary contraction in the male subjects. While there is evidence from single fibre experiments (Bottinelli et al., 1996) that type II fibres have a high force per unit area, no association between composition and maximal isometric knee extensor force (when normalized to lower limb fat‐free mass) was observed. The lack of association is likely explained by the fact that multiple factors interact to determine maximal strength in vivo (O'Brien, Reeves, Baltzopoulos, Jones & Maganaris, 2010). Interestingly, a component of rate of force development (RTD2/3) during the isometric contraction was related to fibre type composition, but only in the males. Furthermore, this was significantly faster in females compared to the males. This observation is difficult to explain with certainty as the rate of force development is influenced by factors in addition to cellular processes within the muscle, such as neural drive and the stiffness of series elastic components (Maffiuletti et al., 2016). Females tend to have lower tendon stiffness than males, and stiffness has also been shown to decline with age in males, both of which would reduce RTD (Kubo, Kanehisa & Fukunaga, 2003; Narici & Maganaris, 2006; Quinlan et al., 2017), while by contrast exercise training can have the effect of increasing tendon stiffness (Kubo, Kanehisa, Miyatani, Tachi & Fukunaga, 2003; Reeves, Narici & Maganaris, 2003). As the interaction between inherent aging, training status, sex and tendon stiffness is complex, this may partly explain the sex difference observed.

## 4 CONCLUSION

The current study examined the relationship between the properties of the VL muscle and both age and appropriate relevant physiological indices in a cohort of highly active male and female cyclists aged 55–79 years. Our contention was that these individuals represented a model of inherent healthy, active aging, free from the confounding negative effects of sedentary behaviour. With the exception of capillary density in males, no association between age and any other property of the VL was observed in either males or females, while a number of associations were observed between the properties of VL of the muscle and indices of physiological and exercise training volume. Overall, these data show no age‐related deterioration in selected properties of the VL muscle which are relevant to aerobic function or explosive muscle power, but are more closely related to an individual's level of function irrespective of their age. The data support the view that high levels of exercise training are able to maintain many of the properties of muscle which are negatively affected by aging when it is accompanied by sedentary behaviour.

## 5 EXPERIMENTAL PROCEDURES

### 5.1 Ethical approval

Prior to participation, written informed consent was obtained from all subjects. Procedures were approved by the National Health Service Wandsworth Research Ethics Committee (reference number 12/LO/0457) and conformed to the Declaration of Helsinki. All human tissues were collected, stored and analysed in accordance with the Human Tissue Act.

### 5.2 Design

A cross‐sectional study evaluating physiological function in a group of amateur nonelite cyclists aged 55–79 was previously conducted (Pollock et al., 2015). Data were obtained from a subgroup of subjects, who at the time of the original study had a muscle biopsy taken. Of 125 participants, 60 males and 30 females agreed to the biopsy procedure (Table 4). For full details of subject selection, see Pollock et al. (2015). Briefly, subjects were healthy, as defined by Greig et al. (1994), 55‐ to 79‐year‐old amateur endurance cyclists. The primary inclusion criteria were that males could cycle 100 km in under 6.5 hr and females 60 km in under 5.5 hr and that this had been done twice in the previous 3 weeks. On average, the subjects of the current cohort had been active cyclists for 26 ± 2 years. All testing was performed over 2 days.

**Table 4:** Anthropometric characteristics of the subjects of the present study and of the entire cohort of master cyclists from the original study

	Current subjects		Entire cohort	
	Male (*n* = 60)	Female (*n* = 30)	Male (*n* = 84)	Female (*n* = 41)
Age (years)	64.5 (0.8)	62.0 (1.1)	64.6 (0.7)	62.1 (0.9)
Height (m)	1.77 (0.01)	1.65 (0.01)^a^	1.77 (0.01)	1.64 (0.01)
Mass (kg)	75.6 (1.1)	60.4 (1.2)^a^	75.4 (1.0)	60.0 (1.0)
Body Fat (kg)	16.0 (0.6)	17.3 (0.8)	15.9 (0.5)	17.2 (0.7)
FFM (kg)	59.7 (0.7)	43.0 (0.8)^a^	59.5 (0.7)	42.9 (0.7)
Lower Limb FFM (kg)	19.9 (0.3)	14.2 (0.3)^a^	19.8 (0.2)	14.1 (0.3)
Dominant Leg FFM (kg)	10.0 (0.1)	7.2 (0.2)^a^	10.0 (0.1)	7.1 (0.1)

FFM, fat‐free mass.

Data are mean (± SE).

a Significantly different from males (*p* < .05).

### 5.3 Body composition

A stadiometer and balance beam scale were used to determine height and mass. Dual‐energy X‐ray absorptiometry (DXA, Hologic Discovery A; Hologic, Bedford, MA, USA) was performed to determine whole‐body and appendicular fat and fat‐free mass.

### 5.4 Muscle characteristics

#### 5.4.1 Muscle fibre type and size

Following administration of local anaesthetic (2% lidocaine), a muscle sample was obtained from the VL using the Bergström needle technique, with applied suction. Part of the sample for histological analysis was mounted in an embedding medium and frozen in liquid nitrogen‐cooled isopentane, while another part was immediately frozen in liquid nitrogen for protein analysis. Biopsies were stored at −80°C until analysis was performed. A cryostat chilled to −20°C was used to cut serial sections (10 μm) which were mounted and stained for myofibrillar ATPase activity. Sections were incubated at a pH of 10.2, 4.6 and 4.3, after which fibres were classified as either type I, IIa, IIc (i.e. hybrid fibres co‐expressing I+II) or IIx. Due to the minimal number of IIx fibres identified (on average 2% with one‐third of subjects having no identifiable IIx fibres), IIa and IIx fibres were combined in a single type II category. On average, one type IIc fibre per subject was found, and therefore, type IIc fibres were excluded from further analysis. Muscle fibre cross‐sectional area (CSA) was determined from areas in good transverse section photographed with a known magnification, calibrated and measured using a digitizing tablet and imagej software (v 1.45k). The percentage area of the muscle sample, used as an estimate of the whole muscle taken up by type I and II fibres, was calculated by multiplying the percentage distribution of each fibre type by its mean CSA and then dividing by the total measured area of both fibre types. The mean (SD) number of muscle fibres used to determine fibre proportions and fibre CSA was 245 ± 79 and 93 ± 36, respectively.

#### 5.4.2 Muscle capillarization

Capillary identification was performed on sections that were fixed in paraformaldehyde (4%), air‐dried and incubated at room temperature with primary antibodies directed against platelet endothelial cell adhesion molecule (PECAM‐1 (aka CD31), dilution 1:500; Cell Signaling Technology, MA, USA) and BA‐F8 (dilution 1:30; DSHB, University of Iowa, USA) to stain endothelial cells and muscle fibres containing myosin heavy chain (MHC) I, respectively. After incubation for 48 hr, slides were washed with PBS (5 × 2 min). Appropriate secondary antibodies were applied: goat anti‐mouse IgG1 Alexa Fluor 488 and goat anti‐rabbit IgG Alexa Fluor 594 (dilution 1:1,000 and 1:1,500, respectively; Invitrogen, CA, USA). After a final wash, slides were mounted with a coverslip using gold antifade mountant with DAPI (Cell Signaling Technology). Staining procedures resulted in endothelial cells stained in green, MHC‐I red and nuclei blue. All images were captured digitally using fluorescence microscopy (ApoTome; Carl Zeiss, Germany). Image processing was performed using AxioVision Software (Carl Zeiss) with subsequent quantitative analysis performed using imagej software. Capillary‐to‐fibre ratio (C/F) was determined by identifying a region of each section, which only included whole muscle fibres and was free from holes or other irregularities. To adjust the C/F for capillaries located on the outside edge of this region, the number of capillaries on the outside edge was divided by 2 before being added to the number of capillaries located on the inner region (Jacobs et al., 2016). This capillary number was divided by the total number of fibres in the region to determine the C/F. Also, the average number of capillaries contacting a fibre (CC) and the average number of capillaries contacting specifically type I or II fibres (CCI and CCII, respectively) were calculated. Finally, capillary density (CD) was determined in the area used for C/F estimation with a normalization factor applied so that results could be expressed as CD per mm^2^.

#### 5.4.3 Mitochondrial protein

Protein content of mitochondrial complexes I–V was obtained from frozen VL muscle which was homogenized using FastPrep‐24TM5G (Millipore, QuickPrep setting, 5 cycles), with the addition of 100 μl ice‐cold lysis buffer (50 mm Tris, pH 7.5, 250 mm sucrose, 1 mm EDTA, 1 mm EGTA, 1% Triton X‐100, 1 mm NaVO4, 50 mm NaF, 0.10% DTT and 0.50% protease inhibitor cocktail). Total protein content was derived from a Bio‐Rad DCTM protein assay (Lot# 500‐0113) using FLUOstar Omega at absorbance 750 nm. Laemmli sample buffer was added prior to loading equal amounts of sample (19.95 μg protein) onto SDS polyacrylamide gels (0.75 mm thickness, 12.5% acrylamide), which were subjected to a constant current of 23 mA/gel for ~45 min at room temperature.

Following electrophoresis, proteins were transferred to a pure nitrocellulose blotting membrane (PALL^®^; Pall Corporation, Life Sciences) at 100 V for 1 hr, with the addition of transfer buffer (0.4 mm Tris base, 1.73 mm glycine) and ice. Following blocking in 3% milk in TBS‐T (Tris‐buffered saline+0.1% Tween), the membranes were incubated overnight at 4°C with total OXPHOS human WB antibody cocktail (Abcam, ab110411), diluted 1:1,000. This was followed by washing in TBS‐T and incubation with secondary antibody (rabbit anti‐mouse IgG, Pierce #31457, diluted 1:10,000). Immobilon Western chemiluminescent HRP substrate (Merck Millipore) was used to quantify protein content after IgG binding and visualized on a G:BOX Chemi XT4 imager using genesys capture software (Syngene UK, Cambridge, UK). Quantification of bands was corrected to the actin band derived from the Ponceau S stain, performed following protein transfer, and by the loading control for each gel.

### 5.5 Physiological measures

For full details of each of the remaining physiological indices measured, see Pollock et al. (2015). Brief details are given below.

#### 5.5.1 Cardiopulmonary exercise testing

Maximal oxygen uptake (VO_2max_) and ventilatory threshold (VT) were determined during a continuous progressive exercise test on a cycle ergometer (Lode Corival; Lode, Groningen, the Netherlands), during which breath‐by‐breath O_2_ and CO_2_ concentrations and volume of expired air were continuously recorded (Oxycon Pro; CareFusion, UK). After a 3‐min warm‐up, cycling at 50 W, power output was continually increased (1–2 W every 3–5 s customized to each subject so that maximal effort would be achieved within 10–12 min) until the subject could no longer continue despite strong verbal encouragement. VO_2max_ was determined as the greatest O_2_ uptake achieved over a 20‐s period at the end of the test. VT was determined using a combination of the ventilatory equivalent method and v‐slope method. VO_2max_ and VT are reported as millilitres of O_2_ uptake per kilogram of fat‐free mass per minute.

#### 5.5.2 O_2_ uptake kinetics

O_2_ uptake kinetics were measured using the same set‐up as for the VO_2max_ testing with a square‐wave protocol conducted twice. Each test began with subjects cycling at 20 W for 6 min followed by a step increase in power output, to that which elicited 80% of the VO_2_ recorded at VT, lasting 6 min. A custom‐written program was used to analyse the O_2_ uptake data that were collected to allow determination of the time constant (τ) of the response to the step increase in power output. This was performed in a similar manner to that described by Rossiter et al. (2002) with full details of the data handling reported in Pollock et al. (2015).

#### 5.5.3 Knee extensor strength

Maximal voluntary contractions (MVCs) of the dominant leg knee extensors were performed in a custom‐built dynamometer with the subjects seated upright, arms folded and the knee in 90° of flexion. Their lower leg, ~3 cm proximal to the ankle, was strapped to a padded steel brace attached to a strain gauge, with the distance from the knee joint centre of rotation to the ankle brace recorded. Subjects performed six MVCs during which force was developed as quickly as possible with the contraction held for 3–4 s. Visual feedback of the force trace was provided in addition to strong verbal encouragement. The maximum torque recorded across all six MVCs was determined and normalized to the fat‐free mass of the dominant limb. In addition, the maximum rate of torque development (RTD) was determined from the slope of the torque–time curve normalized to the relative peak torque in a manner similar to that described by Aagaard, Simonsen, Andersen, Magnusson and Dyhre‐Poulsen (2002). RTD, expressed as a %MVC/s, was calculated from the onset of contraction to one‐sixth, one‐half and two‐thirds of MVC. The onset of contraction was determined from the point where torque exceeded 2.5% of MVC.

#### 5.5.4 Peak explosive power

Peak explosive power (PP), recorded during a 5‐s sprint on a custom‐built inertial testing stationary cycle (Pearson, Cobbold & Harridge, 2004), was determined. Subjects performed a 5‐min warm‐up cycling at 50 W. Three maximal sprints were performed from a starting position where the crank for the dominant leg was vertical at the top of the pedal stroke. An inertial load of 0.325 kg/m^2^ was used for testing. The peak power and velocity at peak power (Vopt) achieved during each sprint were calculated offline using a custom‐written program (Matlab R2013a; The MathWorks, USA). The greatest PP recorded was normalized to the fat‐free mass of the lower limbs and used for subsequent analysis.

### 5.6 Statistical analysis

Statistical analysis was performed using ibm spss statistics v21 (Chicago, IL, USA). The Kolmogorov–Smirnov test was used to assess the normality of the data. For normally distributed data, the relationship between age and the muscle characteristics was assessed by Pearson's correlation coefficient, while for data that were not normally distributed, Kendall's tau correlation was performed. The same statistical tests were used to determine whether any relationship existed between the muscle fibre characteristics and indices of muscular function/cardiorespiratory fitness, and only those relationships that were found to be statistically significant are reported. Relationships between age and indices of muscular function/cardiorespiratory fitness have previously been reported (Pollock et al., 2015). An alpha level of .05 was used for all analyses. All values are presented as mean ± SE unless otherwise stated. When discussing *r*‐values, the following criteria were used to indicate the strength of the association: *r* = .8–1, very strong; *r* = .6–.79, strong; *r* = .4–.59, moderate; *r* = .2–.39, weak; and *r* = 0–.19, very weak.

## CONFLICT OF INTEREST

None declared.

## AUTHOR CONTRIBUTIONS

SH, JL, NL and RP contributed intellectually to the study design. SH took the muscle biopsy samples. RP conducted all physiological testing and data analysis. RP, AR, LD, ND and KN performed ATPase histochemistry/immunohistochemistry analysis and data interpretation. KO and AP performed mitochondrial protein content analysis and data interpretation. SH, RP and NL wrote the manuscript. All authors reviewed and edited the manuscript.
